# Synergistic anticancer effects of nanocarrier loaded with berberine and *miR-122*

**DOI:** 10.1042/BSR20180311

**Published:** 2018-06-27

**Authors:** Lei Li, Xiao Li, Xin Huang, Weilai Jiang, Letian Liu, Chunning Hou, Yanqing Yang, Lei Zhang, Xiaodan Zhang, Longwei Ye, Jie Yuan, Guolin Li, Haiming Sun, Limin Mao

**Affiliations:** 1 Department of Preventive Dentistry, The First Affiliated Hospital of Harbin Medical University, 23 Youzheng Street, Nangang District, Harbin 150001, People’s Republic of China; 2 Department of Oral and Maxillofacial Surgery, The First Affiliated Hospital of Harbin Medical University, 23 Youzheng Street, Nangang District, Harbin 150001, People’s Republic of China; 3 Dental Department, Heilongjiang Electric Power Hospital, Xiangjiang Road, Xiangfang District, Harbin 150030, People’s Republic of China; 4 Laboratory of Medical Genetics, Harbin Medical University, Harbin 150081, People’s Republic of China

**Keywords:** berberine, miR-122, nanocarrier PC, OSCC

## Abstract

We introduced polyethyleneimine (PEI)-cholesterol (PC) as a nanocarrier incorporating berberine (BER) and *miR-122* for the treatment of oral squamous cell carcinoma (OSCC). BER was stabilized by incorporating PC to form *ber*-PC. *Ber*-PC was further electrostatically complexed with *miR-122* to yield *mr-ber-*PC for the co-delivery of BER and *miR-122. mr-ber-*PC treatment dramatically decreased the level of invasion and migration of OSCC cells compared with single drug treatments. The present study suggested that PC could be a multifunctional nanocarrier for the co-delivery of anticancer drug BER and *miR-122* to significantly increase the anticancer therapeutic effects.

## Introduction

Oral squamous cell carcinoma (OSCC), also defined as head and neck squamous cell carcinoma (HNSCC) [1], is one of the most malignant cancers [2]. Epidemiological statistics show that approximately 100000 people worldwide die from OSCC and its associated diseases each year [3]. It is well-known that OSCC is a multicausal disease, current researches indicate that tobacco and alcohol are important factors involved in its occurrence and development. Similarly, chronic inflammation and human herpes virus infection are also closely related to OSCC [4,5]. In addition, susceptibility due to genetic and environmental factors are main etiologies [6]. Patients with OSCC often have metastases, high recurrence rates, and poor prognosis [7]. Therefore, system efficient therapy of OSCC is still a great challenge [5].

Berberine (BER) is a perspective chemotherapeutic agent for the treatment of head and neck squamous carcinomas [8]. As an isoquinoline alkaloid of the *Berberis* species, BER shows potent antimicrobial and antiviral activities [9]. However, due to its low local concentrations, short residence time, and poor intestinal absorption, oral administration of BER is limited [10]. To overcome these problems, it is essential to develop an efficient drug delivery system for BER.

miRNA is a type of non-coding RNA that is approximately 18–23 nt [11]. miRNA genes are often located in tumor-associated genomic regions. These regions are prone to amplifications or deletions and are expressed differently in different tumors [12]. Current evidence has indicated that miRNA could target regulated specific biological characteristics of cancers, including proliferation, apoptosis, metastasis, and metabolism [13–15]. Amongst various miRNAs, *miR-122* is associated with metabolism, anti-inflammatory, and antitumor activities. It is involved in the regulation of a variety of cancers, such as those in the liver, breast, and gallbladder [16–18]. Thus, miRNA-targetted therapies are potential treatments for cancers.

In recent years, great strides have been made in the development of polymeric nanoparticles for pharmaceuticals [19], especially in drug delivery systems. Polymeric nanoparticle-based drug delivery systems are highly viable and versatile for targetted cancer therapy [20].

The molecular origin of cancer has been elucidated at the genetic level. As an emerging technology, gene therapy could treat these disorders by replacing the genetic defects permanently or transiently with exogenous nucleic acids [21]. However, the efficient delivery of nucleic acids remains quite a challenge. Compared with viral vectors, such as adenoviruses and lentiviruses, non-viral gene delivery vectors are considered safe, although they are less efficient [22]. Natural or synthetic polymeric nanoparticles, on the other hand, show great promise because they could be designed to improve both the safety and efficacy of drug delivery.

In our study, we chose polyethyleneimine (PEI)-cholesterol (PC) as a nanocarrier. PC was incorporated with BER and *miR-122* to yield *mr-ber-*PC. Our results demonstrated that *mr-ber-*PC could improve the stability of BER during delivery and promote the delivery efficiency of *miR-122*, which has shown significant inhibitory effects on the invasion and migration of OSCC CAL-27 cells.

## Experimental

### Synthesis of cholesterol-acryloyl and PEI-chol

Cholesterol (7.72 g, 20 mmol) and triethylamine (3.03 g, 30 mmol) were dissolved in DCM (60 ml) in a 100-ml round bottle flask. Acryloyl chloride (2.7 g, 30 mmol) was added into the solution dropwise with stirring. The reaction was conducted for 3 h at room temperature. The mixture was washed with DI water (30 ml) twice and saturated in brine (20 ml). The collected organic phase was dried with anhydrous Na_2_SO_4_ and the solvent was removed by rotary evaporation. The crude paste-like product was purified by silica gel flash chromatography and recrystallized in methanol. The white product was dried under vacuum at room temperature and stored at –20°C*.* In a 20-ml vial, 4 μmol PEI was reacted with 16 μmol cholesterol-acryloyl (chol-acry) in CHCl_3_ (5 ml) and stirred at room temperature for 4 days.

### Synthesis of *ber*-PC

*Ber*-PC was synthesized as follows: PC (100 mg) was dissolved in 5 ml DMSO. BER (10 mg) was dissolved in 5 ml DMSO and gradually added to the PC solution with 8 ml deionized water. The solution was stirred for 1 h. The mixture was then placed in a dialysis bag with molecular weight cut-off as 3500 Da to dialyze against deionized water to remove low molecular weight impurities and unreacted PC. The water was changed every 4 h.

### Synthesis of the nanocarriers loading with miRNA

The miRNA was hsa-*miR-122-5p* and the concentration of miRNA was 10 g/ml. The volume ratio of *ber-*PC (or PC) solution to miRNA solution was 1/1. The *mr-ber-PC* (or *mr-*PC) solution was then obtained. The solution was swirled strongly for 15 s and left at room temperature for 30 min.

### Calculation of drug loading content and drug loading efficiency

One hundred milligrams of PC and 10 ml of BER dissolved in 5 ml DMSO, stirring for 4 h at room temperature. Then 8 ml DI water was added at the speed of one drop/2s to the 2 ml solution and continued to stir for 1 h. The mixture was put into the dialysis bag (MWCO = 3.5 kDa) and dialyzed in the DI water for 24 h to remove the solvent and excess BER. DI water was replaced every 4 h. *miR-122* (10 g/ml) and the *ber*-PC solution were mixed according to the volume ratio of 1/1 and it was strongly vortexed for 15 s then placed at room temperature for 30 min. Then *mr-ber-*PC solution was obtained. Part of *mr-ber-*PC solution was lyophilized for subsequent experiments.

One milliliter of *mr-ber-*PC solution was lyophilized and weighed to calculate the concentration. Then a certain amount of *mr-ber-*PC was dissolved in DMSO and measured the absorbance at 500 nm by UV-Vis spectrometer. The BER content in *mr-ber*-PC was calculated by using the standard curve of pure BER DMSO solution. The formula for drug loading efficiency (DLE) and drug loading content (DLC) are as follows:

DLE(%)=weight of loaded drug/weight of drug in feed×100

DLC(wt %)=weight of loaded drug/weight of polymer×100

### Measurements

The surface potential and particle size distribution and stability of the nanoparticles were measured by Dynamic Light Scattering Apparatus. The concentration of the nanoparticle solution was diluted to 0.1 mg/ml by Milli-Q ultrapure water. A constant temperature was maintained for 20 min before each test. Samples from each group were measured three times to obtain the means.

### TEM observation

The morphologies of the micellar nanoparticles were observed by TEM. Samples were prepared as follows. A drop of micellar nanoparticle suspension (0.5 mg/ml) was added to the surface of 230-mesh number copper net containing carbon film. The acceleration voltage was 200 kV during TEM.

### Cell culture

CAL-27 cells were cultivated in Dulbecco’s modified Eagle’s medium (DMEM) (HyClone, Logan, UT, U.S.A.) containing 10% FBS (HyClone Logan, UT, U.S.A.), and 1% penicillin–streptomycin (Beyotime Biotech, Haimen, China) at 37°C under a humidified atmosphere of 5% CO_2_.

### Cell counting kit-8 assay

*In vitro* cell proliferation was measured using cell counting kit-8 (CCK-8; Beyotime) according to the manufacturer’s instructions. Cells were seeded in 96-well plates at a density of 3 × 10^3^ cells/well. Immediately after treatment for 24 h, 10 μl CCK-8 was added to each well. Cells were then incubated for 2 h at 37°C. The absorbance of each well was measured at 450 nm and repeated three times.

### Real-time PCR

Quantitative real-time PCR was used to determine the levels of *miR-122* and housekeeping gene *U6*. Total RNA samples were extracted from CAL-27 cells using the TRIzol reagent (TaKaRa, Ohtsu, Japan). Transcription Master Kit (Toyobo, Osaka, Japan) was used to reverse transcribe total RNA to cDNA. Real-time PCR was performed with a ABI 7500 Fast Real-time PCR system (Applied Biosystems, CA, U.S.A.) using SYBR Green I (Toyobo, Osaka, Japan). Results were conducted in triplicate in at least three independent experiments.

### Agarose gel electrophoresis

*miR-122* and *mr-ber-*PC were incubated in DMEM (50% FBS) under 37°C. The concentration of miRNA was 10 μg/ml, and samples were taken at intervals of 2, 12, 24, and 48 h. Each solution was then added to 5× loading buffer, mixed, and loaded on to 1% agarose containing Ethidium Bromide (EtBr) (0.5 μg/ml). Electrophoresis was run under 60 V for 30 min. The results were observed with a gel imager.

Configuration of 1% agarose gel: 1 g agarose was added to 100 ml TE buffer (Tris/HCl-EDTA, pH 8.0) and heated in a microwave oven. After it was dissolved, EtBr solution was added at a final concentration of 0.01% of 0.5 μg/ml. The agarose solution was poured into the plastic tank. The gel was ready for electrophoresis after it cooled to room temperature.

Buffer of sample 5×: 0.6 ml Tris/HCl solution (1 M, pH 6.8), 5 ml glycerol (50%), 0.5 ml mercaptoethanol, and 1 ml Bromophenol Blue (1%) were mixed. Then, 2 g SDS was added into the mixture before Milli-Q water was added; the total volume was 10 ml.

### Transwell assay

Transwell assay Matrigel (Corning Life Sciences, New York, NY, U.S.A.) was dissolved at 4°C overnight and diluted by four-fold by serum-free DMEM medium (HyClone). Thereafter, 40 μl diluted Matrigel was gently added to each upper chamber (Corning). Care was taken not to cause bubbles and to ensure that the Matrigel spread evenly to cover the micropores at the bottom of the chamber. Chambers were incubated at 37°C for 4 h. Cell suspension (200 μl) with a density of 2.5 × 10^5^ cells/ml was added to the upper chamber and 600 μl DMEM with 10% FBS (HyClone) was added to the lower chamber. After 48 h, five fields were randomly chosen to calculate the cell numbers. The experiment was performed in triplicate.

### Scratch test

Cell mobility was determined by scratch assays. Scratch test lines were drawn behind the 12-well plate (0.5 cm/per line). Cells were incubated at a density of 3 × 10^4^ cells in the 12-well plates. When the cells reached 80–90% confluence, the medium was removed and a scratch was drawn with a 100-μl pipette tip. Images were captured and the length of the scratch represented the ‘initial’ size of the scratch for that sample. We then transfected the cells. After 48 h of incubation, the cell movements in the scratches were observed and photographed. Scratch closure was quantitated using ImagePro Plus 6 software by measuring the width of the scratch.

### Flow cytometry

OSCC cells were incubated with PC, *ber*-PC, *mr-*PC, and *mr-ber*-PC nanoparticles for 48 h. Cells were then collected and washed twice with PBS and incubated for 15 min at 37°C. Samples were analyzed using an FACS Calibur instrument (company) to determine the number of cells undergoing apoptosis (see Supplementary material for details.)

## Results and discussion

**Figure 1:**
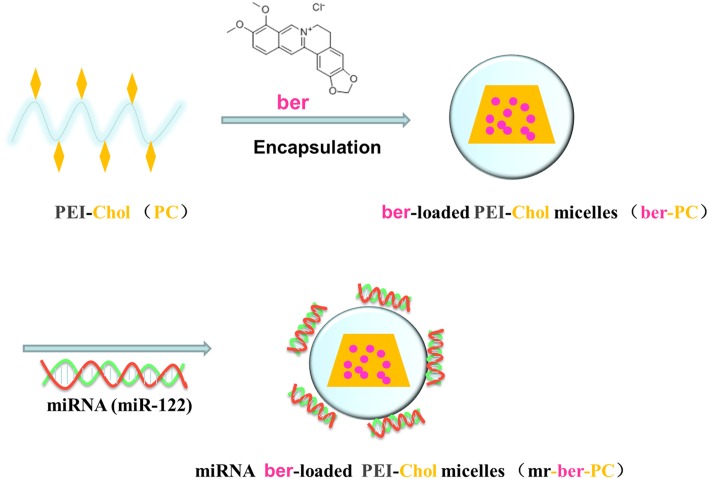
Schematic illustration of the preparation methods of mr-ber-PC BER is entrapped within the PC, while *miR-122* is electrostatically complexed with the positively charged outer shell.

PC was prepared by linear polyethylenimine which was conjugated with chol-acry under a mild condition, while chol-acry was synthesized from cholesterol, trimethylamine, and acryloyl chloride with stirring. Cholesterol was added as a stabilizer for the PC carrier to mediate membrane fusion and cellular internalization of the PC nanocarrier. Furthermore, it improved the stability of nanoparticle dispersion in the medium containing serum by increasing the structural rigidity of the nanocarrier. BER-loaded PC was prepared via the dialysis method. PC can be effectively incorporated BER molecules due to its amphiphilic property. PC was also used as a positively charged shell stabilizer, which resulted in positive charges surrounded the surface of the nanocarrier and enabled electrostatic complexation of the negatively charged *miR-122* with PC (Figure 1). PC can facilitate endosomal escape of the whole drug-loaded nanoparticle via the proton sponge effect. Low-molecular-weight linear PEI (LPEI) was less toxic than the high molecular weight PEI, but less efficient in terms of endosomal escape. Studies have confirmed that endosomal escape can be enhanced, when low-molecular-weight PEI is conjugated with cholesterol [23–25]. Hence, a amphiphilic polymeric nanocarrier (PC) was constructed by conjugating cholesterol molecules to LPEI with low molecular weight, which not only enabled the electrostatic complexation of *miR-122* with PC, but also facilitated the endosomal escape of *miR-122* without obvious toxicity.

PC was synthesized by chol-acry covalent grafting on to LPEI. The grafting molar ratio of cholesterol to LPEI was 3.8%, calculated according to the ratio of the number of grafted cholesterol molecules to LPEI. The DLC and DLE were then determined. The DLC of BER in the nanoparticles was 8.4%, whereas the DLE was 63.0%.

**Figure 2:**
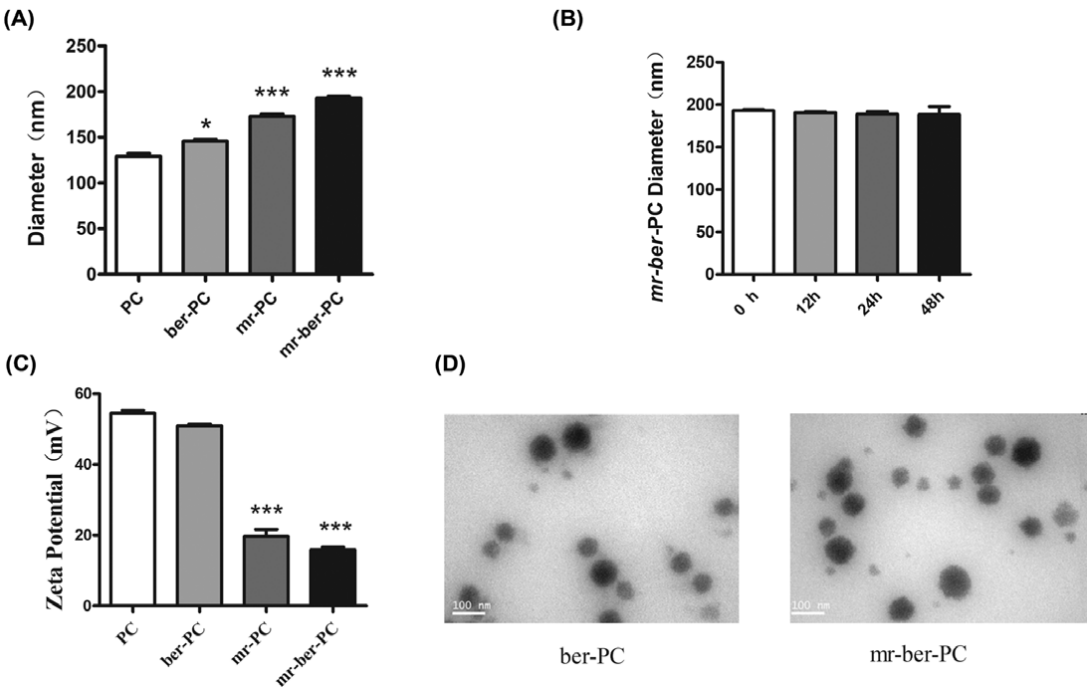
Characterization of prepared nanoemulsions The size and morphologies of polymeric nanoparticles were characterized by DLS and TEM. (**A**) Hydrodynamic diameters of PC, *ber*-PC, *mr*-PC, and *mr-ber*-PC. The results are expressed as mean ± S.D. of triplicate samples. ‘*’ indicates means that are significantly different when compared with the PC group (*P<0.05, ***P<0.001) (**B**) Stability evaluation of *mr-ber-*PC. *mr-ber*-PC did not change significantly in size during incubation at pH 7.4 in PBS at 37°C for 48 h compared with 0 h. (**C**) ζ potentials of PC, *ber*-PC, *mr*-PC, and *mr-ber*-PC. ζ potentials of *mr*-PC, and *mr-ber*-PC are significantly different when compared with the PC group, ***P<0.001 (**D**) TEM images of *ber-*PC and *mr-ber*-PC. The images showed that the diameter of *mr-ber-*PC was significantly larger than that of *ber-*PC, indicating that *miR-122* had successfully loaded into the *ber*-PC.

The size and morphologies of polymeric nanoparticles were characterized by DLS and TEM. As shown in Figure 2, the hydrodynamic diameters of PC (denoted as PC) and BER-loaded PC (denoted *ber*-PC) were 129.2 ± 5.8 and 145.8 ± 2.7 nm, respectively, in PBS at pH = 7.4. By incorporation of BER into the PC, the hydrodynamic size of polymeric nanoparticles is slightly increased due to hydrophobicity improvement. The PC and *ber*-PC complexed with *miR-122* were denoted *mr*-PC and *mr-ber*-PC, respectively. The hydrodynamic diameter of *mr*-PC was 173.1 ± 4.0 nm and that of *mr-ber*-PC was 192.9 ± 4.7 nm (Figure 2A). *miR-122* might surround on the surface of PC and *ber*-PC via electrostatic interaction and increase the diameter of nanoparticles up to 40 nm. Importantly, *mr-ber*-PC did not change significantly in size during incubation at pH = 7.4 in PBS at 37°C for 48 h compared with 0 h and exhibited excellent dispersion stability (Figure 2B). The ζ potential of the complex of *ber*-PC with *miR-122* decreased from 51.0 ± 0.9 to 15.9 ± 1.3 mV in PBS (Figure 2C) as the complex of *miR-122* with the carrier was driven by electrostatic forces. The spherical morphology of the drug-loaded PC was observed via TEM (Figure 2D). The results of TEM imaging of *ber-*PC showed that it formed a smooth spherical nanostructure, indicating that the micelle had self-assembled into nanomicelles in aqueous. The images showed that the diameter of *mr-ber-*PC was significantly larger than that of *ber-*PC, indicating that *miR-122* had successfully loaded into the *ber*-PC.

**Figure 3:**
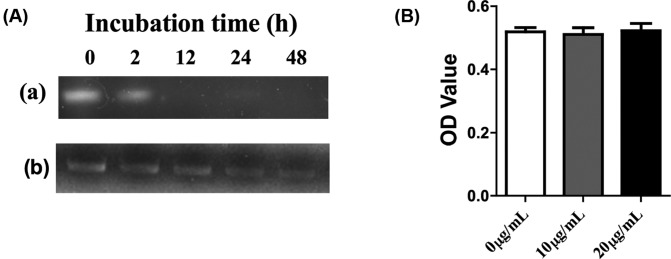
Stability and cytotoxicity of *mr-ber-*PC (**A**) Dispersion stability of (a) *miR-122* and (b) *mr-ber-*PC. Naked *miR-122* degraded completely in 2 h, while *miR-122* in *mr-ber*-PC exhibited excellent stability for nearly 48 h. (**B**) Toxicity of PC via CCK-8 in CAL-27 cells. In different concentrations of *PC*, the viability of the cells was not significantly affected compared with control group (0 μg/ml PC).

The chemical stability of *miR-122* in *mr-ber*-PC against hydrolysis was detected in DMEM at 37°C. At pre-determined time intervals, the samples were collected and analyzed via 1% agarose gel electrophoresis. Naked *miR-122* degraded completely in 2 h, while *miR-122* in *mr-ber*-PC exhibited excellent stability for nearly 48 h (Figure 3A). The cytotoxicity of PC was initially tested in CAL-27 cells, which were treated with different concentrations of PC. After incubation for 48 h, cell viability was measured via CCK-8 assay. The viability of the cells was not significantly affected, which suggests that PC did not exert obvious cytotoxicity (Figure 3B).

**Figure 4:**
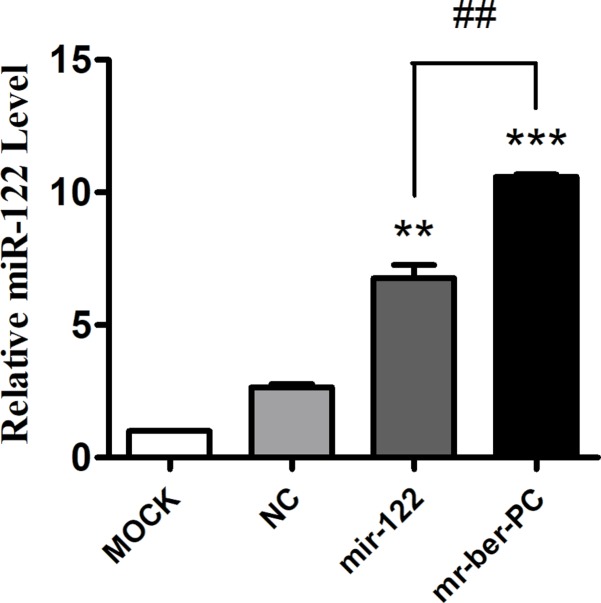
*MiR-122* detection Quantitative real-time PCR was used to detect the *miR-122* level in cells. Compared with NC group, the *miR-122* mRNA expression level in *miR-122* group and *mr-ber*-PC group were both significantly improved. At the same time, it is worth noting that the *miR-122* mRNA expression level in the *mr-ber*-PC group was higher than that in the *miR-122* group. The results are expressed as mean ± S.D. of triplicate samples. ‘*’ indicates means that are significantly different when compared with the NC group, ‘^#^’ indicates means that are significantly different when compared to the *miR-122* group (**P<0.01, ***P<0.001, ^##^*P*<0.01; *n*=3.)

The advantage of *mr-ber*-PC compared with *miR-122* was confirmed by quantitative real-time PCR to detect the *miR-122* levels in cells. Cells were treated with *miR-122* via two methods. NC group and *miR-122* group were transfected with lipo2000 and the corresponding plasmid. Meanwhile, *mr-ber*-PC group was directly added to *mr-ber-*PC. Quantitative real-time PCR was used to detect the *miR-122* level in cells. The results showed that *miR-122* was overexpressed in both the *miR-122* and *mr-ber*-PC group compared with that in the negative control group (NC). Furthermore *mr-ber*-PC was delivered more effectively compared with *miR-122* (Figure 4).

**Figure 5:**
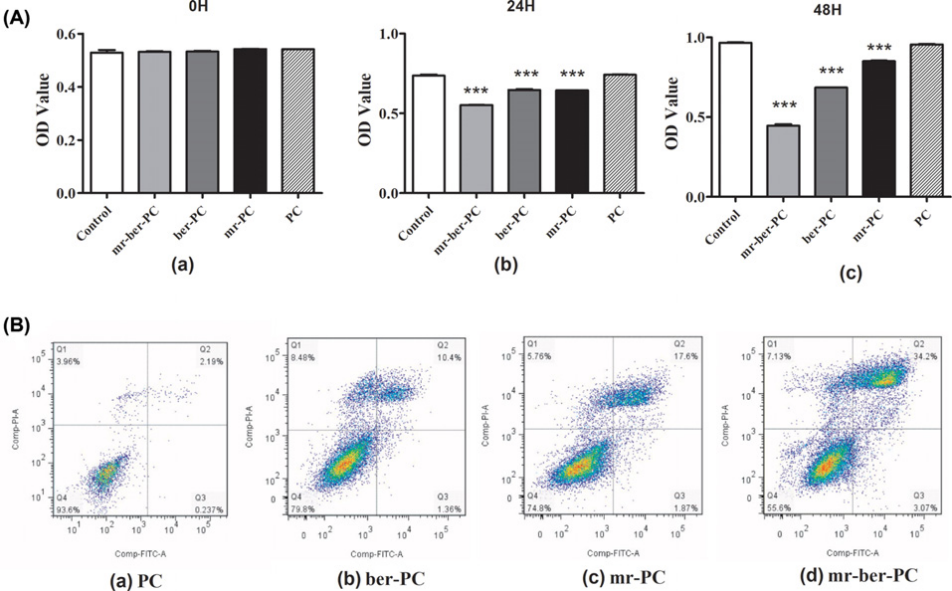

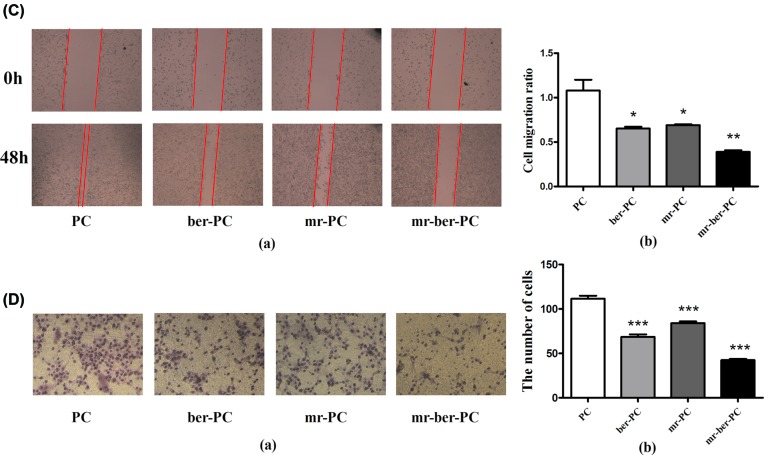
The effects of *mr-ber-*PC on cellular viability, migration, and invasion capacity (**A**) Cell viability was detected via CCK-8. The viability of cells was significantly decreased, which were treated with *mr-ber*-PC, *ber-*PC, and *mr-*PC for 24 or 48 h, compared with PC group. (**B**) A flow cytometry assay was performed to detect apoptosis in cells including four groups, which with different treatments of *ber-*PC, *mr-*PC, and *mr-ber*-PC. Q1, necrotic cells; Q2, late apoptotic cells; Q3, living cells; Q4, early apoptotic cells. The numbers in each panel indicate the percentage of cells present in the quadrant. Compared with NC group, apoptosis was more serious in other groups. (**C**) The migration ability of CAL-27 cells was tested by the Scratch test. The results showed that the migration ability was significant decreased in cells treated with *mr-ber*-PC compared with PC. Meanwhile, *ber*-PC group and *mr*-PC group also showed decreased migration ability compared with PC group. (**D**) Transwell assay was used to detect the migration capacity. The results showed decreased migration ability in cells treated with *ber*-PC, *mr*-PC, and *mr-ber*-PC compared with PC group. And the migration of the PC group was obvious. The results are expressed as mean ± S.D. of triplicate samples. ‘*’ indicates means that are significantly different when compared with the PC group (*P<0.05, **P<0.01, ***P<0.001; *n*=3.)

The cytotoxicity of the co-delivered BER and *miR-122* in CAL-27 cells was evaluated via CCK-8. The viability of cells was significantly decreased, which treated with *mr-ber*-PC, *ber-*PC and *mr-*PC for 24 or 48 h, compared with PC group (Figure 5A). A flow cytometry assay was performed to detect apoptosis in cells subjected to the four different treatments. Compared with NC group, apoptosis was more serious in *ber-*PC, *mr-*PC, and *mr-ber*-PC groups. A relatively high level of apoptosis was observed after *mr-ber-*PC treatment compared with that of the cells by other treatments (Figure 5B).

The effect of *mr-ber*-PC on the migration ability of CAL-27 cells was tested by the invasiveness assay. The cell movements in the scratches were observed. Scratch closure was quantitated by measuring the width of the scratch. The red line is where the cell eventually moves. The results showed decreased migration ability in cells treated with *mr-ber*-PC compared with those treated with PC, *ber*-PC, or *mr*-PC (Figure 5C). The invasive capacity of the cells was evaluated via Transwell assay. CAL-27 cells were treated with PC, *ber*-PC, *mr*-PC, or *mr-ber*-PC separately. And each group of CAL-27 cells was added to the upper chamber and 600 μl DMEM with 10% FBS (HyClone) was added to the lower chamber. After 48 h, we found that the migration of the PC group was obvious. While *ber*-PC and *mr*-PC could decrease invasion, *mr-ber-*PC significantly decreased the invasive capacity compared with PC, *ber*-PC, or *mr*-PC groups (Figure 5D).

## Conclusion

OSCC is the most common malignant tumor in the oral cavity and there are 500000 new cases worldwide each year [26]. A combination of factors such as genetic background and environmental factors can increase susceptibility and lead to the occurrence and development of OSCC [27]. Due to frequent distant metastases, poor prognoses, and high recurrence rates, OSCC has become a critical healthcare problem worldwide [28]. Furthermore, the survival period is unfortunately very short in patients with OSCC [27].

BER is a stable isoquinoline alkaloid and a major bioactive ingredient in Chinese herbal medicine. It has good anti-inflammatory and antitumor effects [29–31] and can inhibit the proliferation and survival of various tumor cells by inducing cell cycle arrest and apoptosis [32–34]. In addition, it has been reported that BER can suppress oral cancer [35].

Many studies have shown that miRNA can regulate the biological characteristics of tumors, including proliferation, apoptosis, metabolism, and so on [13–15]. The number of studies on miRNA-targetted therapies are gradually increasing [36,37]. *miR-122* is reported to be an important target for the treatment of hepatitis C virus (HCV) [38]. In our study, *miR-122* was the target for the treatment of OSCC.

Nanocarriers used for drug delivery are on the nanometer scale and usually approximately 10–200 nm; thus, they can be used to carry a variety of drug molecules. Macromolecules are common materials used to construct nanocarriers. Many types of polymeric drug carriers are currently in clinical trials [39,40].

Cancer is the result of a variety of genetic abnormalities; therefore, the synergistic effect of two or more drugs can achieve better inhibition of tumor growth. This therapeutic strategy has gradually become accepted as an effective way to treat tumors [41,42]. In this study, we designed a nanocarrier containing the synergistic activities of BER and *miR-122* to inhibit OSCC.

We developed a PC-based delivery system and demonstrated that PC can be an effective nanocarrier for the intracellular co-delivery of BER and *miR-122* for anticancer treatments. Moreover, *mr-ber*-PC had significant inhibitory effects on *in vitro* cell invasion and migration.

## Supporting information

**Figure:** Supplementary Material

## Abbreviations

BER: berberine
CCK-8: cell counting kit-8
chol-acry: cholesterol-acryloyl
DLC: drug loading content
DLE: drug loading efficiency
DMEM: Dulbecco’s modified Eagle’s medium
EtBr: ethidium bromide
LPEI: linear polyethyleneimine
OSCC: oral squamous cell carcinoma
PC: polyethyleneimine-cholesterol
PEI: polyethyleneimine
