# Supplementary material for: Synergistic anticancer effects of nanocarrier loaded with berberine and miR-122
Source: Biosci Rep. 2018 Jun 27;38(3):BSR20180311. doi: 10.1042/BSR20180311 (PMC6019385; doi:10.1042/BSR20180311)
Supplement: Supplementary file 1 [file bsr20180311_Supp1.pdf]

Supplementary Material

1. Steps of flow cytometry are as follows:

(1) Cell plating

Logarithmic phase CAL-27 cells were collected and spread onto 6-well plate. The plating density was 5 x 105 cells / well. The plated cells were cultured in the incubator with 5% of CO_2 at 37 ℃ for 24 hr , until the cells were attached.

(2) Drug treatment of cells

The culture medium was pipetted into 6-well plate with transfer pipette, PC, *ber*-PC, *mr*-PC and *mr*-*ber*-PC solution were added to each well and culture for an additional 48 hr.

(3) After the treatment of the cells, the culture medium was discarded, the well was rinsed with precooled PBS for three times and the cells were digested with trypsin, then the trypsin was neutralized with FBS. Initially, 1 mL of cells was removed, centrifuged at 1000 rpm at 4 ℃ for 10 min, discarding the supernatant. Subsequently, 1 mL of precooling PBS was added and gently shaken to suspend the cells. Finally, the cells were centrifugated at 1000 rpm at 4 ℃ for 10 min, that the supernatant discarded. The above experimental steps need to be repeated for three times.

(4) Annexin V-FITC and PI staining

The Annexin V-FITC/PI apoptosis detection kit was used for these analyses (BD reagent company from USA). In brief, the cells were suspended in 200 μL of binding buffer, adding 10 μL of Annexin V-FITC and 5 uL of PI under dark condition and mixed gently. Binding of antibodies and intercalation of PI proceeded at room temperature for 15 min. Finally, 300 μL of binding buffer was added and the sample was analyzed by flow cytometry (BD from USA). At least 1000 cells should be detected in each sample.
